# Dual Immune Modulatory Effect of Vitamin A in Human Visceral Leishmaniasis

**DOI:** 10.1371/journal.pone.0107564

**Published:** 2014-09-30

**Authors:** Bruna L. Lima Maciel, Joanna Gardel Valverde, João Firmino Rodrigues-Neto, Francisco Freire-Neto, Tatjana S. L. Keesen, Selma Maria Bezerra Jeronimo

**Affiliations:** 1 Department of Nutrition, Health Sciences Center, Federal University of Rio Grande do Norte, Natal, Rio Grande do Norte, Brazil; 2 Department of Biochemistry, Bioscience Center, Federal University of Rio Grande do Norte, Natal, Rio Grande do Norte, Brazil; 3 Institute of Tropical Medicine of Rio Grande do Norte, Federal University of Rio Grande do Norte, Natal, Rio Grande do Norte, Brazil; 4 National Institute of Science and Technology of Tropical Diseases (INCT-DT), Salvador, BA, Brazil; McGill University Health Center, Canada

## Abstract

Vitamin A supplementation has shown to prevent mortality by diarrheal and respiratory diseases in several countries. Nevertheless, there are few studies investigating the effect of vitamin A in visceral leishmaniasis (VL), although there are reports of its deficiency in children with symptomatic VL in Brazil and Bangladesh. This study analyzed the effect of vitamin A on a subset of Treg cells and monocytes isolated from symptomatic VL and from healthy children residing in an endemic area for VL in Northeast Brazil. Serum retinol concentrations correlated inversely with IL-10 and TGF-β productions in CD4^+^CD25^high^Foxp3^+^ T cells isolated from children with VL stimulated with leishmanial antigens. *All-trans* retinoic acid *in vitro* induced IL-10 in CD4^+^CD25^high^Foxp3^+^ T cells; IL-10 and TGF-β production in CD4^+^CD25^−^Foxp3^−^ T cells, and IL-10 in monocytes isolated from healthy children. However, the use of *all-trans* retinoic acid together with leishmanial antigens *in vitro* prevented increases in IL-10 production in Treg cells and monocytes isolated from VL children. Strikingly, those results show a potential dual role of vitamin A in the immune system: improvement of a regulatory profile in cells from healthy children after leishmanial stimulation and down modulation of IL-10 in Treg cells and monocytes during symptomatic VL. Therefore, the use of vitamin A concomitant to VL therapy might be useful in improving recovery from disease status caused by *Leishmania infantum* infection and warrants additional study.

## Introduction

*Leishmania infantum* infection is endemic in Brazil and can evolve with a large spectrum of clinical outcomes, ranging from asymptomatic, self-healing infection, to disease, visceral leishmaniasis (VL). Symptomatic VL is characterized by fever, hepatosplenomegaly, hypergammaglobulinemia, weight loss and susceptibility to other infections such as viral, bacterial or fungi, with about 5–10% of mortality, even with treatment [1], [2]. The determinants of Leishmania infection outcomes, either disease or self-resolution of the infection are not clearly understood, but could be influenced by interactions of the parasite and the immune response of the host and by environmental factors such as comorbidities [3] and nutritional status [4]–[6].

Although interferon-gamma (IFN-γ) has been found in blood and bone marrow [7], [8] of VL patients, classically symptomatic VL has been characterized by failure of peripheral blood mononuclear cells to proliferate or to produce IFN-γ in response to *Leishmania* antigens. In addition, VL patients present skin anergy to intradermal leishmanial antigens, which is usually reversed months after clinical cure [9]–[11]. Consistently, interleukin-10 (IL-10) production, is considered a hallmark of symptomatic VL [10], [12]–[14]. Studies have demonstrated that macrophages may be important sources of IL-10 during VL [15], [16]. Recently, regulatory T (Treg) cells have been implicated in IL-10 production and CD4^+^ suppression in visceral leishmaniasis [13], [17]–[19]. In human VL, Nylén *et al.* [13] found CD4^+^CD25^−^Foxp3^−^ Tr1 cells as the major source of IL-10. However, Rai *et al.* [18] showed enrichment of IL-10 in CD4^+^CD25^+^Foxp3^+^ cells isolated from bone marrow of human VL cases. In addition, TGF-β, which produced by Treg cells, is important during VL. Gantt *et al.* [20] found high levels of TGF-β in bone marrows of VL patients, and showed that TGF-β had deactivating effect on macrophage function, both *in vitro* and *in vivo* [21].

Although much has been studied to understand the human immune response against *Leishmania* infection, little is known about the determinants of the immune responses during the disease. In addition to the complex interactions between the parasite and the human immune response [22], [23], other variables such as nutritional status might influence the outcome of *Leishmania* infection [24], [25]. It is known that chronic infections slowly deplete serum retinol, which leads to reduced immunity and increased susceptibility to further infections, as viral, bacterial or fungal, in a poorly understood cyclical relationship, which could be a risk factor for VL development [26], [27]. Although we [6] and others [6], [28] have found vitamin A deficiency in VL patients, the mechanism by which vitamin A influences the immune response to an intracellular pathogen, as *Leishmania*, is not understood. Studies have consistently demonstrated that *all-trans* retinoic acid, the main active vitamin A metabolite in the cells, enhances TGF-β induction of Foxp3 in Treg cells [29]–[31]. Intriguingly, vitamin A deficiency in mice seems to be associated with elevated IL-10 competent Treg cells [32].

Experimental immunization studies have also shown that *all-trans* retinoic acid promotes antibody responses to T cell-dependent antigens [33], [34]. A mice model has shown that vitamin A deficiency enhances the development of IL-10 Th2 secreting cells and reduces Th1 development [35]. *All-trans* retinoic acid can suppress Th17 development [31] by increasing TGF-β signaling and reducing the expression of the IL-6 receptor [36].

Although large cohort trials have shown that vitamin A supplementation decreases global mortality by 30% [37], most of the work attempting to understand the effect of vitamin A on specific T cells subsets is experimental, and its mechanisms of action remain poorly understood. Moreover, there are few published data in animal models or in human *Leishmania* infections linking the role of vitamin A in the immune response to the parasite [38], although vitamin A deficiency is prevalent in areas endemic for VL [6] and some of these areas tend to adopt the World Health Organization's protocol for vitamin A supplementation [39].

This study aimed to analyze the effect of vitamin A in the response against *Leishmania* infection in a subset of Treg cells and monocytes isolated from children with symptomatic VL, prior to treatment, and from healthy children potentially exposed to *L. infantum* by virtue of sharing the same environment of a VL case. We found increased IL-10 production by CD4^+^CD25^high^Foxp3^+^ T cells, IL-10 and TGF-β production by CD4^+^CD25^−^Foxp3^−^ T cells and IL-10 by monocytes isolated from healthy children after *in vitro all-trans* retinoic acid stimulus. Interestingly, the combination of *all-trans* retinoic acid and leishmanial antigens *in vitro* prevented increases in IL-10 production in Treg cells and monocytes isolated from VL children, indicating a potential immune modulatory effect of vitamin A during *L. infantum* infection.

## Materials and Methods

### Study groups, inclusion and exclusion criteria

Subjects were recruited from an open cohort of subjects residing in a *L. infantum* endemic area of Northeast Brazil, as previously described [40] and also subjects under treatment for VL from July 2009 to December 2012. These later cases were from the same neighborhood described previously [40]. Twenty-six children were recruited for the present study. Children were grouped as follows: 1) Children with symptomatic visceral leishmaniasis (VL) prior to treatment (n = 10), and 2) Healthy children with no apparent signs of *L. infantum* infection, but residing in the endemic area and having a relative with visceral leishmaniasis (n = 16). Symptomatic VL cases were recruited in the hospital, whereas the healthy children were enrolled at their household, during a follow up visit after the VL subject was discharged from the hospital.

Inclusion criteria for each group were as follows:

1. Active VL: children with current, prior to treatment, symptomatic VL, with the diagnosis of disease status confirmed by presence of Leishmania in bone marrow aspirates and/or positive rK39. The clinical and laboratorial criteria for symptomatic disease were defined as intermittent fever for more than 3 weeks, hepatomegaly and/or splenomegaly, hypergammaglobulinemia, pancitopenia, low platelets, hematocrit and hemoglobin levels. Hemogram analyzes were done 0–1 day before immunological assessment in referral hospitals;

2. Healthy children with no apparent signs of *L. infantum* infection (Endemic controls): children with negative skin delayed type hypersensitivity (DTH-), Montenegro skin test, and negative anti-*Leishmania* antibodies, who were a contact of a VL case. *Leishmania* antigens were a water-soluble fraction of promastigote cultures obtained from an isolate of a human subject with VL (*Leishmania infantum,* IOC 3052). The skin test was placed after blood sample collection. rk39 was used to determine presence of antibodies against Leishmania [41]. Hemogram analyses and clinical evaluation were performed in this group and they presented no alterations indicative of diseases. In addition, those children presented no clinical signs of other infections such as diarrhea, coughing or fever.

### Ethical considerations

The study protocol and informed consents were reviewed and approved by the Federal University of Rio Grande do Norte Ethical Committee. The certificate of ethical approval is CAAE 0087.0.051.000-09 and is available at the http://aplicacao.saude.gov.br/plataformabrasil/login.jsf. The consent form was signed by a parent or a legal guardian of the participants.

### Determination of *Leishmania* infection

Two ELISA assays using soluble lysate antigens (SLA) and rK39 protein as source of antigens were used, as previously described [41]. Briefly, wells of ELISA plates (Costar) were coated with 200 ng of soluble *Leishmania* antigens (SLA) or 50 ng of rK39. The SLA was obtained from Leishmania infantum promastigote grown in culture. Leishmania isolate was obtained from a bone marrow aspirate of a human subject with symptomatic VL. The isolate was shown to be *L. infantum* by isoenzymes. Each serum sample was assayed in triplicate. Each plate included negative control sera from unexposed Brazilians and positive control sera from patients with documented VL. The A_405_ was determined using a Titertek Multiskan ICN - plate reader. The cut-off was the mean plus 3 standard deviations absorbance of negative control sera. The cut off values for the antibodies were established from the analysis of 32 blood samples collected from individuals not exposed to the endemic area.

Montenegro skin test was performed using 25 µg of *Leishmania amazonensis* proteins (Centro de Produção e Pesquisa de Imunobiológicos, Secretaria de Saúde, Paraná, Brazil) injected intradermally. Skin tests were read after 48–72 h, measured in two perpendicular directions using the ball-point pen method [42]. A positive test result was defined if the mean of the two induration measurements was greater than 5 mm [43].

### Serum retinol determination

Serum retinol was determined by high pressure liquid chromatography (HPLC) by a modified procedure, using fasting serum samples [44]. Briefly, 500 µL of ethanol 95% containing 1 µg/mL of retinyl acetate as internal standard was added to 400 µL of sera for protein precipitation. Samples were washed, three times, with 500 µL of hexane (Merck, São Paulo, Brazil). In each time, the hexane phase was collected into a new test tube. These hexane layers were evaporated under nitrogen gas at 37°C. The resulting extracts were ressuspended in 70 µL of methanol (Merck, HPLC grade) and 20 µL was injected in the HPLC system. Mobile phase was a 100% methanol at a 1.0 mL/min flow and detection was at 325 nm. A reverse phase C18 Microsorb MV column, with 4.6×150 mm, 100 Å pore and a MPLC NewGuard Holder complete C18 (reverse phase), 3,2×15 mm guard column were used.

### Peripheral blood mononuclear cells separation and culture

Peripheral blood mononuclear cells (PBMC) were isolated from heparinized venous blood after centrifugation over a Ficoll-Paque Plus gradient (GE Health Care Life Sciences). PBMC were suspended in RPMI-1640 (Sigma-Aldrich) supplemented with 10% of inactivated autologous sera, in presence of antibiotics (penicillin 200 U/mL and streptomycin, 0.1 mg/mL) and maintained in 96-well plates, 2.5×10^5^ cells in 200 µL, per well, for 20 h at 37°C and 5% CO_2_. Five different conditions were used in the cultures as follows: media alone, SLA (10 µg/mL), *all-trans* retinoic acid (0.25 nM), SLA (10 µg/mL) + *all-trans* retinoic acid (0.25 nM) and concanavalin A – ConA (10 µg/mL). *All-trans* retinoic acid concentration was determined by titration under same culture conditions, with cells stained for anexin V and Propidium Iodide (PI) and then analyzed by flow cytometry. The concentration of 0.25 nM was chosen for the best FSC and SSC PBMC profile and low anexin V and PI staining.

### Cell staining and Flow cytometry analyses

During the last 6 h of culture, brefeldin A (1 µg/mL) (eBioscience) was added to improve cytokine detection. The cells were then washed and stained for surface markers, and fixed using 2% formaldehyde (Sigma-Aldrich). Fixed cells were permeabilized and stained using anticytokine monoclonal antibodies. Immunoglobulin control antibodies and a control of unstimulated PBMC were included in all experiments [45].

Monoclonal antibodies directly conjugated with fluorocromes were: anti-CD4 APC-Cy7 (BD Biosciences), CD25 PE-Cy7 (eBioscience), CD14 PerCP (eBioscience), Foxp3 Alexa fluor 488 (BD Biosciences), TGF- β1 PerCP (R & D Systems), IL-10 APC (BD Biosciences) and IL-17F PE (eBioscience).

**Figure 1:**
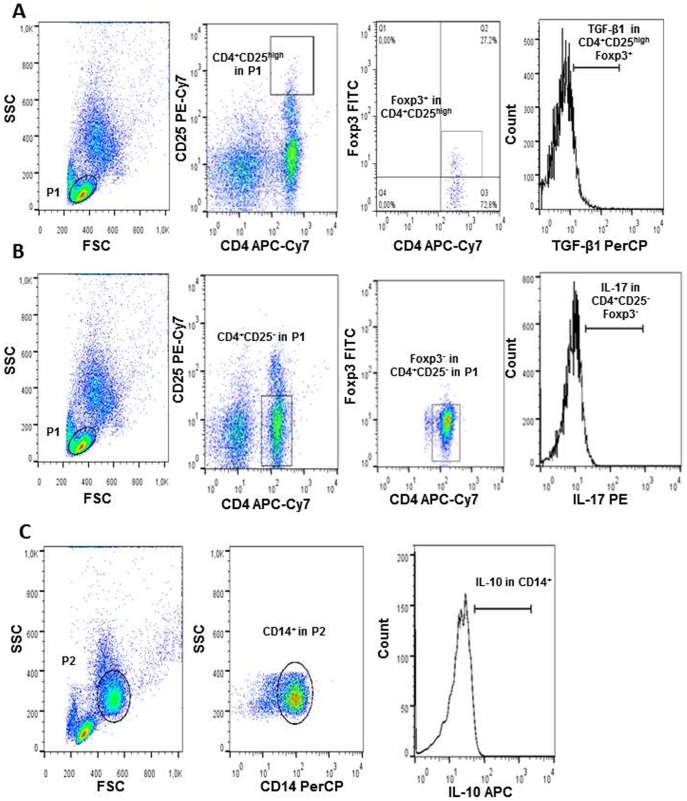
Representative graphs of Treg cells and monocytes. A. Flow cytometry dot plots represent lymphocytes region (P1), frequency of CD4^+^CD25^high^Foxp3^+^ T cells and cytokine analysis in these cells (TGF-β1 shown as example). B. Flow cytometry dot plots represent lymphocytes region (P1), frequency of CD4^+^CD25^−^Foxp3^−^ T cells and cytokine analysis in these cells (IL-17 shown as example). C. Flow cytometry dot plots represent monocytes region (P2), and IL-10 analyzed in CD14^+^ monocytes. Graphs are examples of 20 h cultures of PBMC without any stimulus.

Preparations were acquired on BD FACSCanto II (BD Biosciences) and 30,000 events were acquired for the analysis. Data was processed using FlowJo software, version 7.6.5 (Tree Star Inc, Ashland, OR, USA). Specific gating strategies to select Treg CD4^+^CD25^high^Foxp3^+^, CD4^+^CD25^−^Foxp3^−^ and monocytes (CD14^+^) subsets are presented through representative density plots which are shown in Figure 1. Treg cells were stained and analyzed for TGF-β1, IL-10 and IL-17, while monocytes were stained for IL-10.

### Statistical analysis

Kolmogorov-Smirnov test was used to determine the normality of all the variables in study. To detect differences within the groups for age and retinol Mann-Whitney Test was used and these variables are presented as median (interquartile range – IQR). To determine whether serum retinol was associated with cytokine production in different cell subsets after SLA stimulus correlation analysis was done using Spearman correlation coefficient (r^2^). Mann-Whitney test was used to determine whether there were differences in Foxp3 and cytokine production, in culture, under different stimuli between endemic controls to VL subjects; or Wilcoxon matched pairs test to compare cultures stimulated and non-stimulated within studied groups. A significant difference was accepted when the p value was less than 0.05. Statistical analysis was performed using the GraphPad Prism software, version 5.

## Results

### Patients with symptomatic visceral leishmaniasis present low serum retinol

Of the 26 children studied, 10 had active VL, prior to treatment; and 16 were healthy children, but a household contact of a VL case. These latter children were both negative for anti-Leishmanial antibodies and Montenegro skin test [median induration (IQR) 0.0 (0.0) mm]. All children with VL had parasitological confirmation of *Leishmania* through a positive bone marrow aspirate and they also had positive anti-*Leishmania* antibodies. The two groups were homogenous, with similar age and gender distribution (p>0.05) (Table 1). Vitamin A status was significantly lower (Mann-Whitney test, p = 0.013) in children with symptomatic VL (Table 1), with median serum retinol of 20.34 (13.17) µg/dL in the VL group and 25.69 (3.97) µg/dL in the endemic control group (Table 1).

**Table 1:** Characteristics of the studied groups by age, gender, anti-*Leishmania* antibodies, Montenegro skin test response and serum retinol concentrations.

		Studied Groups		*p* * Value
	Total	controls	Active VL	
Parameters	n = 26	n = 16	n = 10	
Age (yr) Median (IQR)	8.38 (6.52)	8.82 (6.26)	7.99 (7.85)	0.815
Gender N (%)				
Male	14 (53.8)	11 (68.8)	3 (30.0)	0.063
Positive anti-rK39 N (%)	10 (38.5)	0 (0.0)	10 (100.0)	<0.0005
Positive anti-SLA N (%)	8 (30.8)	0 (0.0)	8 (80.0)	<0.0005
Montenegro response (mm of induration) Median (IQR)	0 (0.0)	0 (0.0)	φ	-
Serum retinol (µg/dL) Median (IQR)	25.23 (8.77)	25.69 (3.97)	20.34 (13.17)	0.013

*For continuous variables Mann-Whitney Test was used; for categorical variables Fisher's exact test was used. ^φ^Not done in this group.

### Serum retinol inversely correlated with IL-10 and TGF-β1 in CD4^+^CD25^high^Foxp3^+^ T cells

**Figure 2:**
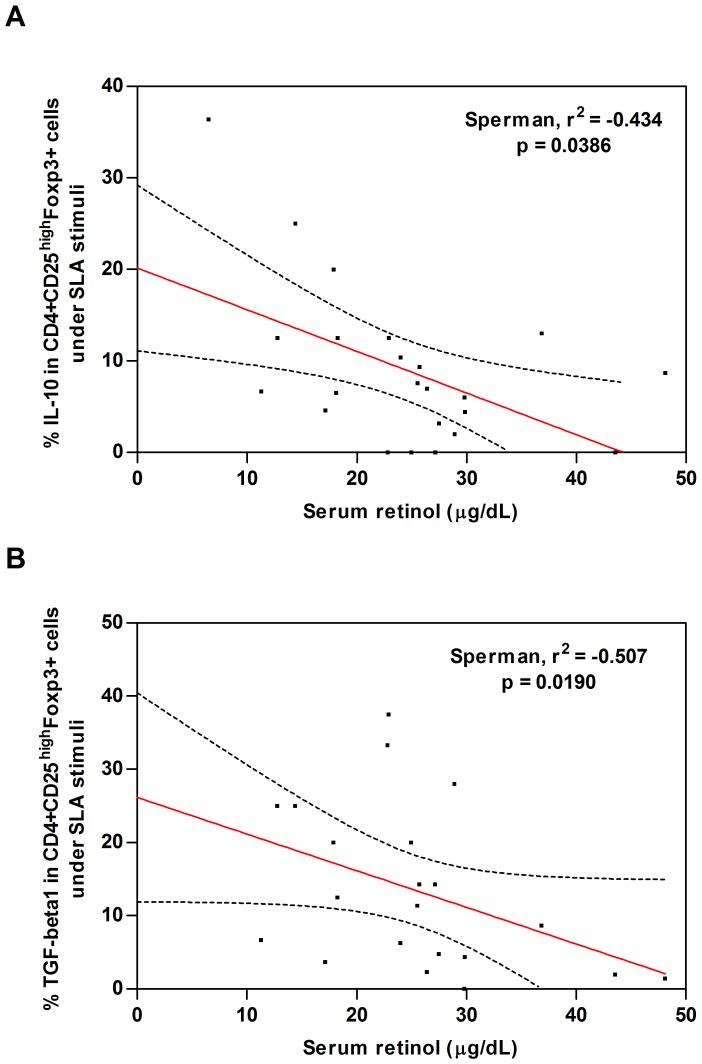
Negative correlation between serum retinol and IL-10 and TGF-β1 production by CD4^+^CD25^high^Foxp3^+^ T cells. **A**. Represents a negative correlation between serum retinol and IL-10 production in CD4^+^CD25^high^Foxp3^+^ T cells after SLA stimulus. **B**. Represents a negative correlation between serum retinol and TGF-β1 production in CD4^+^CD25^high^Foxp3^+^ T cells after SLA stimulus. Correlation analyzes were performed in VL cases (n = 10) and endemic controls (n = 16), using Spearman correlation coefficient.

Since CD4^+^CD25^−^Foxp3^−^ T cells, CD4^+^CD25^high^Foxp3^+^ T cells and monocytes have been shown to be important source of IL-10 in VL [9], [12], [14], we evaluated whether these cells function change with respect to the serological level of vitamin A. As shown in Figure 2A, we found a significant negative correlation between serum retinol and IL-10 production in CD4^+^CD25^high^Foxp3^+^ T cells after SLA stimuli (Spearman, r = −0.434, p = 0.0386). We also found a significant negative correlation between serum retinol and TGF-β1 production in CD4^+^CD25^high^Foxp3^+^ T cells (Spearman, r = −0.507, p = 0.0190, Figure 2B).

No significant correlation was found between serum retinol and IL-10, TGF-beta1 and IL-17 production in CD4^+^CD25^high^Foxp3^+^ T cells in medium (not shown, Spearman, p>0.05). No correlation was found for the same cytokines in the medium or after SLA stimulation for CD4^+^CD25^−^Foxp3^−^ T cells and IL-10 in CD14^+^ cells (not shown, Spearman, p>0.05).

### CD4^+^CD25^high^Foxp3^+^ T cells are lower in VL patients and increased by antigen and *all-trans* retinoic acid stimuli

**Figure 3:**
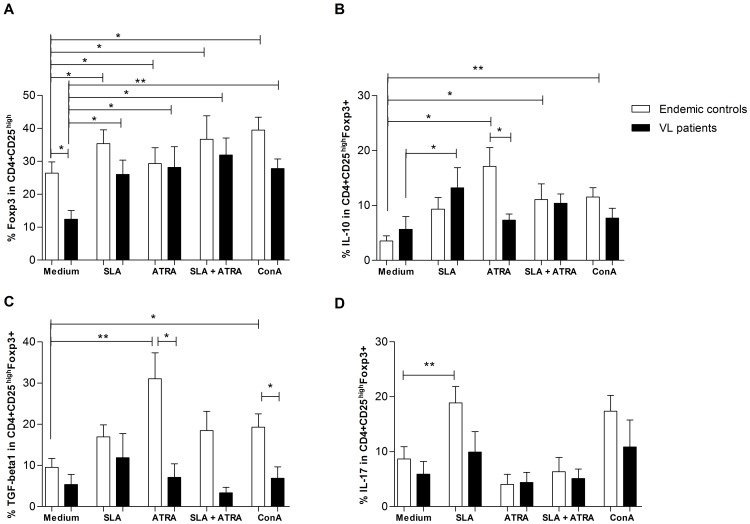
Cytokines expression in CD4^+^CD25^high^Foxp3^+^ T cells in response to different stimuli. **A**. Lower frequency of CD4^+^CD25^high^Foxp3^+^ T cells in VL patients when compared to endemic controls and its increase after all stimuli. **B**. IL-10 production in CD4^+^CD25^high^Foxp3^+^ T cells after tested conditions. **C**. TGF-β1 production in CD4^+^CD25^high^ Foxp3^+^ T cells after tested conditions. **D**. IL-17 in CD4^+^CD25^high^ Foxp3^+^ T cells after tested conditions. Mann-Whitney test was used to compare endemic controls to VL patients. Wilcoxon matched pairs test was used to compare cultures stimulated and non-stimulated within the studied groups. * p<0.05; ** p <0.01. Subjects tested: endemic controls (n = 16) and VL patients (n = 10). Bars represent median and IQR. ATRA  =  all-trans retinoic acid. SLA  =  Soluble *Leishmania* antigens (SLA). ConA  =  Concanavalin A.

Since vitamin A status inversely correlated with CD4^+^CD25^high^Foxp3^+^ T cells cytokine response after SLA stimulus, we further looked into whether the cytokine response of CD4^+^CD25^high^Foxp3^+^ T differed after different stimuli between the two groups (Figure 3). VL children presented significant lower CD4^+^CD25^high^Foxp3^+^ T cells when compared to endemic controls (Mann-Whitney, p = 0.011). However, overall after stimuli with all tested conditions, cells from the endemic controls and VL subjects had increased frequency of CD4^+^CD25^high^Foxp3^+^ T cells and there was no difference between VL patients and endemic controls (Figure 3A). This result potentially indicates that, *in vivo*, *Leishmania* antigens and vitamin A induce expansion of CD4^+^CD25^high^Foxp3^+^ T cells.

### *All-trans* retinoic acid induces distinct cytokine production in CD4^+^CD25^high^Foxp3^+^ T cells from visceral leishmaniasis subjects

CD4^+^CD25^high^Foxp3^+^ T from VL subjects had increased IL-10 production after SLA stimulation, whereas endemic controls had not (Figure 3B). Interestingly, *all-trans* retinoic acid significantly increased IL-10 in cells isolated from the endemic controls, but the same effect was not observed in VL subjects. However, *all-trans* retinoic acid used in combination with SLA abrogated the effect of IL10 production in CD4^+^CD25^high^Foxp3^+^ T isolated from VL subjects (Figure 3B). However, for endemic controls, *all-trans* retinoic acid + SLA stimulated IL-10 production. Similarly, *all-trans* retinoic acid significantly induced TGF-β1 in CD4^+^CD25^high^Foxp3^+^ T cells isolated from the endemic controls (Wilcoxon,  = 0.037), but not for VL subjects (Figure 3C). CD4^+^CD25^high^Foxp3^+^ T cells from controls after SLA stimulation had significantly more IL-17 production (Wilcoxon, p = 0.009), but not in CD4^+^CD25^high^Foxp3^+^ cells from VL subjects (Figure 3D). *All-trans* retinoic acid whether alone or in combination with SLA produced no effect on IL-17 production in CD4^+^CD25^high^Foxp3^+^ T cells from VL patients or endemic controls (Figure 3D). The cytokine response observed after *all-trans* retinoic acid or SLA stimulation in CD4^+^CD25^high^Foxp3^+^ is more likely driven by specific antigens in the SLA preparation and *all-trans* retinoic acid, as can be observed by comparison of the response to *all-trans* retinoic acid and SLA with Concanavalin A.

### *All-trans* retinoic acid decreases IL-17 production by CD4^+^CD25^−^Foxp3^−^ T cells from VL subjects

**Figure 4:**
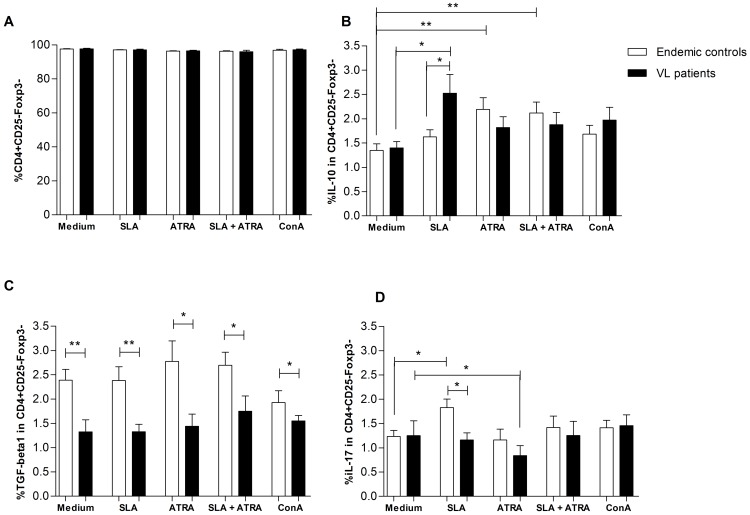
Cytokines expression in of CD4^+^CD25^−^Foxp3^−^ response to different stimuli. **A**. Similar frequency of CD4^+^CD25^−^Foxp3^−^ T cells in studied groups and conditions. **B**. IL-10 production in CD4^+^CD25^−^Foxp3^−^ T cells after tested conditions. **C**.TGF-β1 production in CD4^+^CD25^−^Foxp3^−^ T cells after tested conditions. **D**. IL-17 production in CD4^+^CD25^−^Foxp3^−^ T cells after tested conditions. Subjects tested, endemic controls (n = 16) and VL patients (n = 10). Mann-Whitney test was used to compare endemic controls to VL patients. Wilcoxon matched pairs test was used to compare cultures stimulated and non-stimulated within the studied groups. * p<0.05; ** p<0.01. Bars represent median and IQR. ATRA  =  all-trans retinoic acid. SLA  =  Soluble *Leishmania* antigens (SLA). ConA  =  Concanavalin A.

CD4^+^CD25^−^Foxp3^−^ T cells frequency was similar between the studied groups (Figure 4A), but cells isolated from VL patients responded to SLA stimulation with significantly more IL-10 production (Figure 4B). After *all-trans* retinoic acid stimulation, an increase in IL-10 production by CD4^+^CD25^−^Foxp3^−^ T cells was seen only in cells isolated from endemic controls, in presence or absence of SLA stimuli (Figure 4B). However, CD4^+^CD25^−^Foxp3^−^ did not increase the production of TGF-β1 in response to any of stimuli tested (Figure 4C). Surprisingly, CD4^+^CD25^−^Foxp3^−^ T cells isolated from VL subjects showed lower production of TGF- β1 when compared to endemic controls (Figure 4C).

It was interesting to note that after SLA stimulation CD4^+^CD25^−^Foxp3^−^ T cells of endemic controls significantly increased IL-17 production (Wilcoxon, p = 0.013), but not cells from VL subjects (Figure 4D). *All-trans* retinoic acid reduced IL-17 production in cells from VL (Wilcoxon, p = 0.039). No effect was seen when adding *all-trans* retinoic acid + SLA in IL-17 production by cells from cases or controls (Figure 4D).

### *All-trans* retinoic acid prevented the increase in IL-10 produced by monocyte from VL subjects

**Figure 5:**
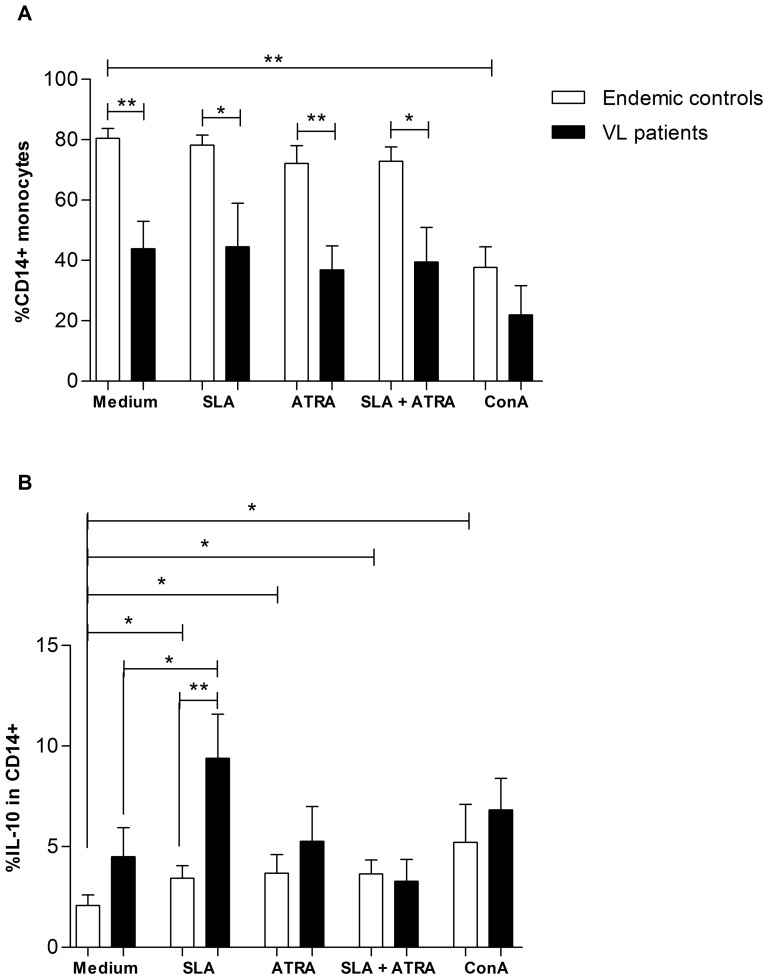
IL-10 expression in CD14^+^ monocytes after different stimuli. **A**. Significant reduced frequency of CD14^+^ monocytes in VL patients. **B**. IL-10 production in CD14^+^ monocytes after tested conditions Mann-Whitney test was used to compare endemic controls to VL patients. Wilcoxon matched pairs test was used to compare cultures stimulated and non-stimulated within the studied groups. * p<0.05; ** p<0.01. Bars represent median and IQR. ATRA  =  all-trans retinoic acid. SLA  =  Soluble *Leishmania* antigens (SLA). ConA  =  Concanavalin A.

We further analyzed the effect of the different stimuli on IL-10 production in monocytes. There was a decrease in the frequency of CD14^+^ monocytes in VL subjects (Figure 5A). Moreover, subjects with VL produced significantly higher amounts of IL-10 after SLA stimulus when compared to endemic controls (Mann-Whitney, p = 0.002) or to the medium (Wilcoxon, p = 0.023) (Figure 5B). Interestingly, *all-trans* retinoic acid significantly increased IL-10 production in endemic controls, but the same effect was not observed in VL patients. Once more, the use of *all-trans* retinoic acid + SLA in VL patients prevented the increase of IL-10 seen after SLA stimulation alone (Figure 5B). However, for endemic controls, *all-trans* retinoic acid + SLA stimulated IL-10 production.

## Discussion

Nutrients can influence the immune response [46], [47] and the immune response itself can act synergistically in response to drug treatment during diseases, improving recovery [48]. Therefore, the efficacy of drug therapy could potentially be improved, if used in combination with immunostimulants [49], [50]. Experimental models in VL have shown that antagonizing the effect of IL-10 dramatically enhanced the leishmanial activity of Sb^v^ [51], [52]. Nevertheless, few studies have attempted to use nutrients as immune modulators in VL, although it is well documented that malnutrition increases the risk of developing visceral leishmaniasis [1], [5]. In this study, we showed a correlation of vitamin A status and cytokines in response to *Leishmania* infection. Moreover, we observed a dual effect of vitamin A: in healthy children, it induced a regulatory profile and in subjects with VL it prevented IL-10 increase after stimulation with leishmanial antigens.

Impaired vitamin A status is a characteristic of VL, previously shown by us [6] and others [28]. Our previous study showed, that the low concentrations of serum retinol in VL were not uniquely related to the acute stress response in *Leishmania* infections [6]. To our knowledge, this is the first study in human VL that finds a negative correlation between serum retinol and key cytokines to this disease such as IL-10 and TGF-β (Figure 2). This reinforces the importance of vitamin A as regulatory factor in the immune response that might influence the outcome of *Leishmania* infections.

Stephensen *et al.* [35] also observed that vitamin A deficiency was associated with enhanced development of IL-10 producing T cells. In that study, they were not able to detect whether these cells were Th2 or Treg cells. Nevertheless, the study from Maynard *et al.* [32] found that, during vitamin A deficiency, C57BL/6 mice had increased numbers of competent IL-10 producing Treg cells. Taken together, these results indicate that during vitamin A deficiency Treg cells may be important subsets in IL-10 production. In our study, SLA alone enhanced IL-10 production in cells isolated from VL subjects, which is in accordance to the studies of Rai *et al.* [18] and Nylén *et al.* [13] that found high production of IL-10 in nTreg, Tr1 and monocytes, respectively after *Leishmania* antigen stimulation. However, no significant IL-10 production was observed when *all-trans* retinoic acid and SLA were added. Intriguingly, in cells isolated from the endemic controls, the use of *all-trans* retinoic acid in combination with SLA enhanced IL-10 and TGF-β1 production in CD4^+^CD25^−^Foxp3^−^ T cells and IL-10 in monocytes. These results might indicate a regulatory role for vitamin A in Leishmania infection. Prior studies have exploited this regulatory role of vitamin A, in auto-immune disorders [53], [54].

The role of Treg cells in leishmaniasis remains controversial, although they seem to be important cells in T CD4+ suppression during VL [18]. Despite the ability of Treg cells to suppress excessive immune response against the Leishmania, most of the studies have shown that these cells may promote an excessive regulatory control which allows parasite replication, survival and long term persistence [55]–[58]. Surprisingly, our data clearly demonstrates that Treg cells and monocytes isolated from VL patients have different responses when stimulated with *all-trans* retinoic acid comparing to healthy controls. These results indicate a defect in cell function during *L. infantum* infection. One possibility to explain the lack of response after *all-trans* retinoic acid stimulus in VL is cell exhaustion and this has been successfully demonstrated in T CD8^+^ cells in some studies during VL [59], [60]. In this study, the use of *all-trans* retinoic acid together with leishmanial antigens *in vitro* prevented increases in IL-10 production in Treg cells and monocytes isolated from VL children. Our results illustrate the effects of a single vitamin A metabolite in the balance of the immune response.

Moreover, we found low CD4^+^CD25^high^Foxp3^+^ T cells in PBMC, and this corroborated the finding of Nylén *et al.* in Indian VL [13]. This result may indicate that there is not a failure in the expansion of CD4^+^CD25^high^Foxp3^+^ T cells during VL, but possibly a differential homing of these cells to the sites of infection. Nylén *et al.* [13] saw no increase of these cells in the spleen of humans with VL, but Rai *et al.* [18] observed enrichment of these cells in the bone marrow of subjects with disease.

Interestingly, cells from endemic control showed pronounced IL-17 production after SLA stimulus in CD4^+^CD25^high^Foxp3^+^ and CD4^+^CD25^−^Foxp3^−^ T cells while VL subjects did not. Our data corroborates the results from Pitta *et al.* [62] who found an increased production of IL-17 in PBMC of healthy individuals after *L. donovani* stimulus, signalizing that this cytokine plays a role in the protective response against *Leishmania* infection [61]. However, CD4^+^CD25^−^Foxp3^−^ T cells from VL significantly decreased IL-17 production after *all-trans* retinoic acid stimulus. Nevertheless, our results of *all-trans* retinoic acid reducing the production of IL-17 and stimulating CD4^+^CD25^high^Foxp3^+^ T cell expansion are in accordance to studies which have shown that retinoic acid can stimulate Treg conversion and reduce Th17 conversion in the presence of TGF-β [30], [31], [36], [63]–[65].

*All-trans* retinoic acid effect on cells seems to be dose-dependent. Dawson *et al.* [66] observed that increasing doses of *all-trans* retinoic acid (1, 10 and 100 nM) led to increased IL-5, IL-4 production and decreased IFN-γ by PBMCs. Elias *et al.* [63], observed that in even higher doses of *all-trans* retinoic acid (1 nM, 100 nM and 10 µM) progressively decreased IL-17 production and increased Foxp3 expression in T cells isolated from mice spleen and lymph nodes. A problem in analyzing the effect of different a*ll-trans* retinoic acid concentrations used in these studies is that in none of them, cell viability or cell death data were observed after culture with the different *all-trans* retinoic acid concentrations. Nevertheless, the results from these studies show that *all-trans* retinoic acid effect was more likely linear, once inversion in its effect with increasing doses was not observed.

It has been extensively shown that PBMC from VL patients lack the ability to produce IFN-γ in response to *Leishmania* antigen stimulation [9]–[11], although increased amounts of IFN-γ are found in whole blood cell assays from VL patients [7], [8]. Moreover, it is well known that *all-trans* retinoic acid inhibits IFN-γ gene expression possibly by directly acting on its promoter [66], [67]. In our study, IFN-γ was not analyzed, but subjects had a skin test negative.

Studies in *Leishmania* and other infections have shown that there may be differences in the innate immune responses between males and females [68]–[71]. In addition, there is an increased risk of developing visceral leishmaniasis after age 10 among males, although there is no difference between the rate of *L. infantum* infection between males and females residing in urban endemic area for VL (Monteiro et al, unpublished results). In this study, we only analyzed cells isolated from children and there was no difference in gender distribution among the study subjects. Therefore the question of the impact of gender in the inate immunity needs different design, considering age and sex differences. Surprisingly, our study has found lower levels of TGF-β1 production in CD4^+^CD25^−^Foxp3^−^ T cells of VL patients when compared to endemic controls. This result may be explained by the cell population analyzed, which may not represent the main TGF-β1 producers during VL. Although it is known that TGF- β is high in VL [20], few studies have identified the source of this cytokine production.

Not many studies have addressed the role of nutrients on *Leishmania* infection. Garg *et al.* [38] in a model of hamsters infected with *L. donovani* observed that vitamin A supplements given prophylactically or after infection promoted increased parasite burden. Conversely, our study showed that *all-trans* retinoic acid increased IL-10 and TGF-β1 in Treg and IL-10 in monocytes isolated from healthy children. However, *all-trans* retinoic acid plus *Leishmania* antigens stimulation prevented the increased production of IL-10 after SLA stimulus by cells isolated from children with VL, which may indicate a potential modulatory effect of *all-trans* retinoic acid in cytokine associated to disease.

In summary, our results show that Treg cells and monocytes during VL have different responses to stimuli when compared to cells from healthy controls, indicating an important role of these cells during the disease. Furthermore, we show that vitamin A may present a dual role in the immune response regulation: improvement of regulatory responses in healthy children exposed to *Leishmania* antigens and down modulation of IL-10 expression in Treg and monocytes during VL. Therefore, the use of vitamin A concomitant to VL therapy needs to be evaluated in further studies. Finally, this study reinforces the importance of vitamin A, a single nutrient that comes from the diet, which is a modifiable risk factor, in the immune response to *Leishmania* infection.
