# Correction: Dual Immune Modulatory Effect of Vitamin A in Human Visceral Leishmaniasis

**DOI:** 10.1371/journal.pone.0115080

**Published:** 2014-12-01

**Authors:** The PLOS ONE Staff

There is an error in the citation within the XML version of the article. The correct citation is: Maciel BLL, Valverde JG, Rodrigues-Neto JF, Freire-Neto F, Keesen TSL, et al. (2014) Dual Immune Modulatory Effect of Vitamin A in Human Visceral Leishmaniasis. PLoS ONE 9(9): e107564. doi:10.1371/journal.pone.0107564.
